# Coping With Food Insecurity Using the Sociotype Ecological Framework

**DOI:** 10.3389/fnut.2018.00107

**Published:** 2018-11-15

**Authors:** Wen Peng, Sandro Dernini, Elliot M. Berry

**Affiliations:** ^1^Department of Public Health Nutrition, Medical School, Qinghai University, Xining, China; ^2^Department of Public Health, Amity Foundation, Nanjing, China; ^3^Forum on Mediterranean Food Cultures, Rome, Italy; ^4^Department of Human Nutrition & Metabolism, Braun School of Public Health, Hebrew University-Hadassah Medical School, Jerusalem, Israel

**Keywords:** the sociotype, food insecurity, coping, resilience, ecological model

## Abstract

Ensuring Food Security (FS) for all citizens is a fundamental human right and policy for all countries. Dealing with Food Insecurity (FINS) is a challenge causing stress at many levels—national, household, and individual. The conceptual framework of the Sociotype has been developed as a summary ecological construct to organize the multiple, dynamic, reciprocal inputs from the environment that interact with the genotype to determine the expression of phenotypic behaviors such as coping with stress. The Sociotype ecological framework has three domains—Individual (intra-personal), Relationships (inter-personal, family and community), and Context (environment, national)—and their interactions determine an individual's resilience across the life trajectory from birth to old age. We have applied the principles of the Sociotype to classify both the stresses of, and the coping strategies to, FINS. The stresses of FINS may occur at any place along the FS pathway—Availability, Accessibility, Utilization, and Stability. The elicited coping responses may take place in one or more of the three Sociotype domains. The two processes are inter-related linearly with re-iterative feedback loops such that stress leads to coping responses that may or may not be adequate, thereby requiring modifications in the coping strategies until FS is regained. Resilience is considered to represent long-term coping strategies. The Sociotype framework is both a determinant of, and constant input to, building and strengthening resilience. However, the people with the most problems with FINS are rarely included in these discussions. They are the marginalized members of society: unemployed, homeless, displaced persons, special needs, elderly, single parents, mentally frail, and more. Applying the Sociotype ecological framework for coping with FINS stresses can allow better strategic planning for FS at national, household and individual levels and understanding the interactions between them to reach out to and help these sectors of the population.

## Introduction

The need to eat food is the most important biological drive in all living species. It is even more essential/significant than that the drive to reproduce since when food is scarce, women lose their menstrual cycles and men their libido. If there are not enough energy stores in adipose tissue to maintain a successful pregnancy, then nature will delay the process (1, 2). Therefore, the stresses of food insecurity (FINS) are a major threat to a country and may occur at different levels—national, household, and individual. There are many methodologies and indicators by which to measure food security (FS) and FINS that depend on the analysis of data from primary (expert opinion and community perspectives) and secondary (collected by governments) sources (3–5). However, the people with the most problems with FINS are rarely included in surveys. These are the marginalized members of society: unemployed, homeless, displaced persons, special needs, elderly, single parents, mentally frail, and more. Much more efforts need to be made to reach out to and help these sectors in the population. To try and understand better the stresses of, and coping strategies for, FINS, we wish to introduce a concept called the Sociotype (6–8).

This paper attempts to classify the stresses causing FINS and the coping strategies developed to deal with them according to the Sociotype framework within its three domains. At the outset, the concepts of food security and insecurity are detailed briefly as a background to understanding how the sociotype may interact with them.

## Food security and food insecurity

FS is best considered as a causal, linked *pathway* from production to consumption, through distribution to processing, recognized in a number of domains, rather than as four “pillars” (3)—availability, accessibility, utilization, and stability. The fourth dimension deals with the ability of the nation/community/(household) person to withstand shocks to the food chain system whether caused by natural disasters (climate, earthquakes) or those that are man-made (wars, economic crises). Thus, it may be seen that FS exists at a number of levels. Availability–National; Accessibility–Household; Utilization–Individual; Stability–may be considered as a time dimension that affects all the levels. *All four of these domains must be intact for full food security*.

More recent developments emphasize the importance of sustainability, which may be considered as the long-term time (5^th^) dimension to food security (8). Sustainability involves indicators at a supra-national/regional level of ecology, biodiversity and climate change, as well as socio-cultural and economic factors (9). These will affect the FS of future generations.

FINS on the other hand, will occur when there are problems at any one level in the food production-consumption pathway. The definition of FINS is “whenever the availability of nutritionally adequate and safe foods, or the ability to acquire acceptable foods in socially acceptable ways, is limited or uncertain” (10). FINS as practically measured in the United States, is experienced when there is (1) uncertainty about future food availability and access, (2) insufficiency in the amount and kind of food required for a healthy lifestyle, or (3) the need to use socially unacceptable ways to acquire food. FINS can also be experienced when food is available and accessible but cannot be utilized because of physical or other constraints, such as limited physical functioning by elderly or disabled or underlying chronic disease (11).

However, with the emphasis on health equity, focus should be given to the people under the most disadvantaged conditions. In 2015, some 795 million people worldwide, who were 12.9% of world population, did not have enough food to lead a healthy and active life. The majority lived in developing countries in Asia (511.7 million) and Sub-Saharan Africa (220.0 million) (12). However, the number increased to 815 million in 2016 (13). They are under various natural and man-made stresses such as floods, droughts, conflicts, and wars. They also have urgent demand for better coping strategies for FINS.

FS and FINS are dynamic, reciprocal, and time dependent and the resultant status depends on the interaction between the stresses of FINS and the coping strategies to deal with them. The stresses of FINS may occur at any point along the FS pathway–Availability, Accessibility, Utilization, and Stability. The elicited coping responses may take place at the national, household or individual levels. The two processes are inter-related linearly with re-iterative feedback loops such that stress leads to coping responses that may or may not be adequate, thereby requiring modifications in the coping strategies until FS is regained. Therefore, given the vulnerability of certain population/individuals, FS is a function of the stresses and the coping, which may be tested empirically (14).

## The conceptual framework of the sociotype and its application to coping with food insecurity

The Sociotype is a summary ecological construct to organize the multiple, dynamic, reciprocal inputs from the environment that interact with the genotype to determine the expression of phenotypic behavior, especially coping. It is commonly accepted that a person is the product of his/her genes (genotype, DNA, nature) and the environment (nurture), which together determine his/her observable characteristics and behavior, or phenotype. We have extended the ecological model of Bronfenbrenner (15) and the bio-psychosocial framework of Engel (16, 17) to formulate the concept of the sociotype (6, 7). The Sociotype is a framework synthesis of the factors that determine an individual's resilience across the life trajectory from birth to old age. It is considered to be made up of three domains—Individual (intra-personal), Relationships (inter-personal), and Context. Individual factors include physical and mental health, personality and life philosophy. Relationships cover all interactions within the family, friends and at work. The Context factors include education, employment, socio-economic position and demographics, and the relevant political system (7).

**Figure 1:**
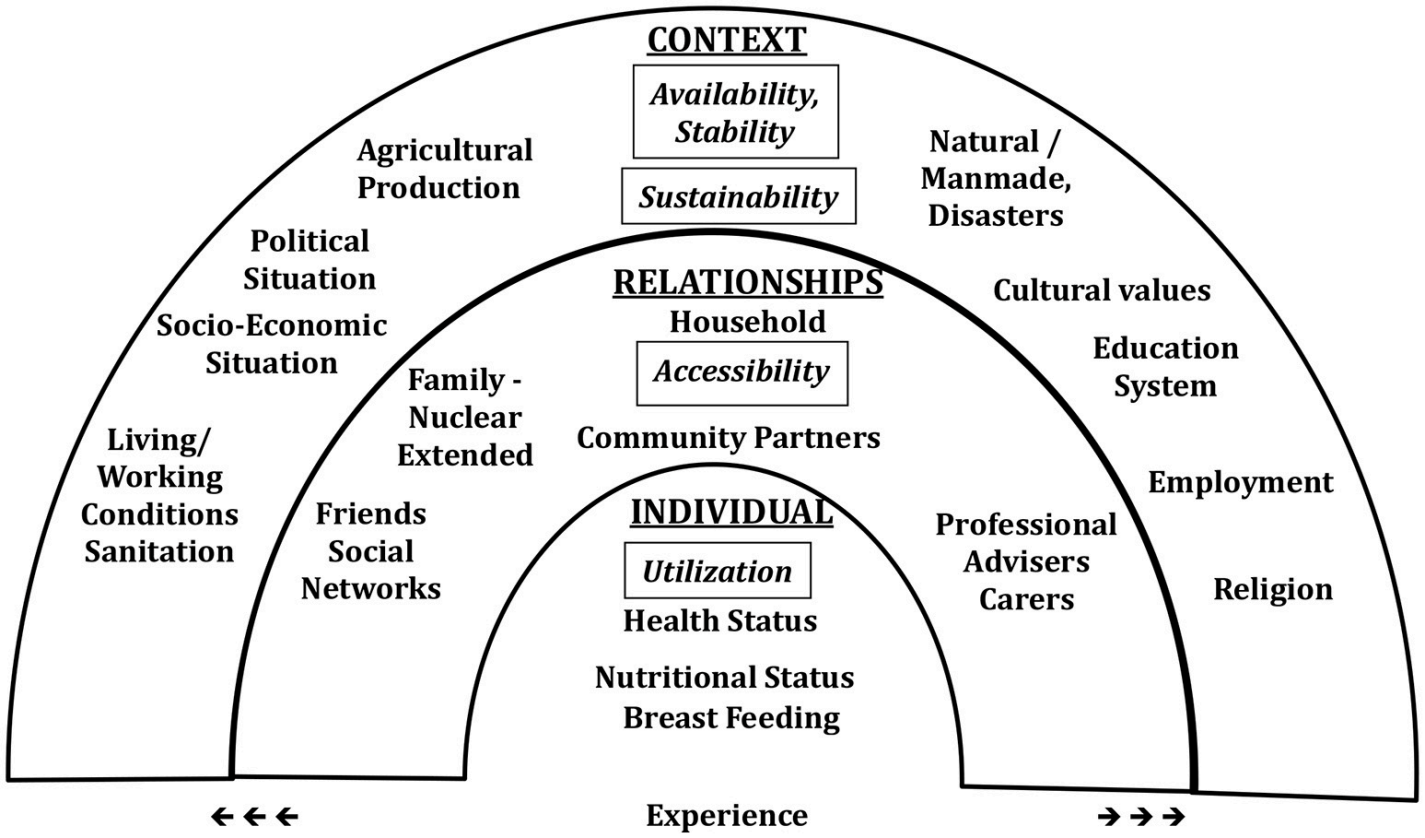
Sociotype ecological framework of some of the elements of the sociotype in relation to the stresses of, and coping strategies for, food insecurity. Italics show the dimensions of food security and sustainability.

The sociotype also incorporates a multi-level perspective and has been developed to highlight the importance of an extended bio-psychosocial perspective, which combined medical reductionism with the psychosocial aspects of the behavioral sciences concerned with “problems of living” (16, 17). The sociotype adds to these intra- and inter-personal inputs a further outer ecological layer concerned with the contextual environmental influences on phenotypic responses. Thus, the sociotype, with its three domains, is a summary ecological construct to organize the multiple, dynamic, reciprocal inputs from the environment that interact with the genotype to determine the expression and behavior of the phenotype throughout life. Advances over the past 50 years have led to a vast literature on the nature and understanding of the human genome, and recent work on epigenetics is beginning to show that environmental influences may also affect the expression of the genotype (18). However, while the inputs of the genotype on the phenotype are relatively stable, those of the sociotype are constantly changing with experience and circumstances. The sociotype defines the intra-personal, inter-personal and extra-personal determinants of behavior. The term was apparently first used by Bogardus (19) to represent the effects of society on behavior in a very general way; however, it was not developed further. Recently, del Moral et al. have also applied the concept of “Sociotype” in mental health and quality of life (20). The current usage positions the sociotype to understand the determinants of an individual's behavior and ability to adapt/cope to life situations generally in health and disease, and more specifically to FINS. The three domains proposed for the sociotype are in line with ecological models for assessing environmental influences on human behavior (21). Figure 1 shows the ecological model of the sociotype in relation to domains of FS and sustainability.

The entries in each of the three domains are not exhaustive and depend on the behavior under study. There is no one “normal” sociotype as each individual's upbringing and personality determine responses to different stress situations (6, 7). The “success” of these behavioral patterns is dependent on the inputs from the sociotype.

The interactions between these three domains will determine how a person will respond cognitively and then cope with any stress such as the life events of marriage, divorce, bereavement, aging, or chronic disease situations such as diabetes and cancer. Recent results from a survey on over 1,200 adult respondents show that coping and mastery skills for life events are independent of age and increase with educational achievements. Of the three Sociotype domains, Individual health was the most important determinant of coping ability. Women valued contextual factors more than relationships whereas in men the opposite was found. Individuals who are less healthy and wealthy have poorer coping scores (8).

A person's Sociotype and that of his family will also be challenged by the stresses of FINS. Similarly, the conceptual framework of the Sociotype could be applied to the household and community as a collective summary of the individual Sociotypes.

## Stresses of food insecurity

To clarify how the Sociotype framework facilitates the understanding of FINS, we first attempt to classify the stresses of, and coping strategies for, FINS. Classification of the stresses of FINS is not exclusive. The same stress may affect several dimensions of FS—Availability, Accessibility, Utilization, and Stability. For example, Nepal's earthquake in 2015 damaged agriculture and road infrastructure, thus affecting both the availability and accessibility dimensions of food security. Breakdown in sanitation and onset of infectious diseases after an earthquake could impair the health status of individuals, thus affecting the individual's utilization of food, which further exacerbates FINS. Stresses at different levels—national, household, individual—may interact. Stresses at higher levels (regional, national) will affect the lower levels (household, individual) of FS. In some cases, if the lower-level stresses are chronic or severe, the impact direction may reverse. For example, the Syrian civil war was a man-made stress to FINS at the national level, which affected millions at the individual level. On the other hand, the refugees from the war fled to neighboring countries including some European countries. The large number of refugees have added additional burden to these countries' existing financial crisis, thus bringing FINS problems to these countries. This is an example how stresses of FINS at the national level expanded to the regional level. To cope with it, monthly food assistance was provided by WFP to more than four million Syrians inside Syria, and food needs of 1.5 million Syrian refugees in neighboring countries were also reached. But these numbers were far away from the numbers who needed help—by the beginning of 2016, 4.6 million Syrian citizens became refugees and some 13.5 million people within Syria needed humanitarian assistance (13). In fact, only minor stresses may have impacts limited to a single dimension or at single level. Therefore, the attempt to categorize the stresses, as shown in Table 1, only enables understanding the stresses of FINS and emphasizing their major impacts. The scale and the chronicity of the stresses are changeable, depending on how effective the coping strategies are.

**Table 1:** Examples of the stresses of acute and chronic food insecurity categorized according to the dimensions of food security and sustainability.

**Chronicity**	**Dimension/level**	**Availability**	**Accessible**	**Utilization**	**Stability**	**Sustainability**
Acute	Global/Regional	1. Regional natural disasters: floods, earthquakes, e.g., 2015 Pacific hurricane season 2. Large- scale manmade disasters: Wars/conflicts, e.g., World War I, II, Vietnam, Rwanda, Bosnia …	1. Economic: food price changes in international trade, e.g., 2007–2008 global food price crisis exacerbated the food insecurity in Sub Saharan Africa		Any acute stress disturbing the production-consumption pathway at any level, e.g., global/regional level: global or regional war; national/subnational level: natural disasters; household level: unemployment; individual level: diseases.	Not applied to acute stresses
	National/sub-national	1. Natural or manmade disaster, e.g., cyclones, Syria, civil war 2. Unstable political conditions, e.g., 1998 Sudan famine	1. Physical: destruction of infrastructure by natural or manmade disasters, e.g., 2008 China Wenchuan earthquake 2011 Japan Fukushima earthquake 2015 Nepal Gorkha earthquake 2011 Syria civil war	1. Breakdown in Sanitation—post natural disaster, e.g., earthquakes, floods	
	Household		1. Economic: food price changes in domestic market; unemployment, 2. Family misfortune e.g., catastrophic expenditure (diseases, accidents), loss of labor force in households		
	Individual			1. Acute diseases: acute infection, etc.	
Chronic	Global/Regional	1. Regional natural disaster: drought, e.g., 2005–2006 West Africa 2. Unfair international trade policies manipulate food prices and jeopardize food security in developing regions (import/export)	1. Economic: increased international crop price damages food security in developing countries, especially those relying on import	Regional epidemics of communicable diseases:Polio in Nigeria, Ebola in West Africa SARS in East Asia	Not applied to chronic stresses.	Sustainability is the ecological long-term dimension of food security, which affects all levels from global to individual. 1. Global Warming Climate change—global /regional extreme weather conditions, increased frequency of natural disasters 2. Urbanization and soil productivity loss—loss of arable land 3. Biofuel in developed countries consumes large amount of crops 4. Increased demand for meat in emerging countries poses stress to environment, e.g., China, Brazil 5. Agriculture subsidies in rich countries made them dump their surplus to developing countries and damage their agricultural sector, e.g., EU dumping to African countries 6. Genetically-Modified-Organism may harm environment and nature, e.g., North America, China
	National/sub-national	1. National-scale natural disaster, e.g., drought, frequent cyclone and floods 2. Inadequacy in food imports, e.g., iron deficiency leads to high prevalence of anemia in Cuba	1. Physical: poor logistic chain/road rail infrastructures, e.g., remote areas 2. Economic: chronic poverty at national/sub-national level 3. Social-culture: gender bias makes girls and women vulnerable to food insecurity	1. Poor sanitation2. Poor health care/education 3. Food safety—pesticides residue, endocrine disrupting chemicals	
	Household		1. Physical: lack of proper transport 2. Economic: poverty at household level 3. Social-cultural: gender inequity jeopardizes the allocation of food to women including pregnant women	1. Improper food conservation, processing and preparation	
	Individual		1. Illness/aging/disability prevents mobility	1. Chronic Illness e.g., HIV/AIDS, diabetes affecting digestion, absorption and utilization of food	

## Coping with the stresses of food insecurity

When FINS stresses occur, a number of compensatory coping mechanisms/strategies come into play (22, 23). No matter at which level the coping occurs, the aim of coping strategies and behaviors is to ensure that FS returns to the household and its individuals (but not necessarily return to the same level as before the stress). In coping with FINS, a number of questions arise in each of the Sociotype domains as detailed in Table 2. The answers to these questions (and there are many more) cannot be easily tackled through randomized controlled trials and require a more qualitative type of methodology (24).

**Table 2:** Questions arising when tackling food insecurity by domains of the sociotype.

**INDIVIDUAL**
How to deal with constant hunger?
How to feed people with limited economic resources in the rural and city context?
How to keep up food quality and prevent malnutrition with limited economic resources?
How to eat previously unacceptable food–culture/religious taboos?
**RELATIONSHIPS (Family, Community)**
How to decide to whom to distribute the food?
How to guarantee the food security of the key population, e.g., pregnant women, children?
How to cope if someone falls ill?
How to borrow or get credit?
**CONTEXT**
How to use the community collective power to get through the “hard” season?
How to prioritize resources under economic hardship?
How aid should be allocated and operated?
What types of help should be arranged?
How to build community resilience?
How markets should play their role?
How financial tools should play their role?

Coping with FINS involves two dynamic, linearly correlated and re-iterative stages—(1) assessments of the stresses and (2) the coping strategies to deal with them. There is a feedback, which monitors the success, or otherwise, of the strategies leading to modifications as appropriate. The reiterative feedback loop assesses how coping will develop/change over time according to the effectiveness of the coping strategies employed.

**Figure 2:**
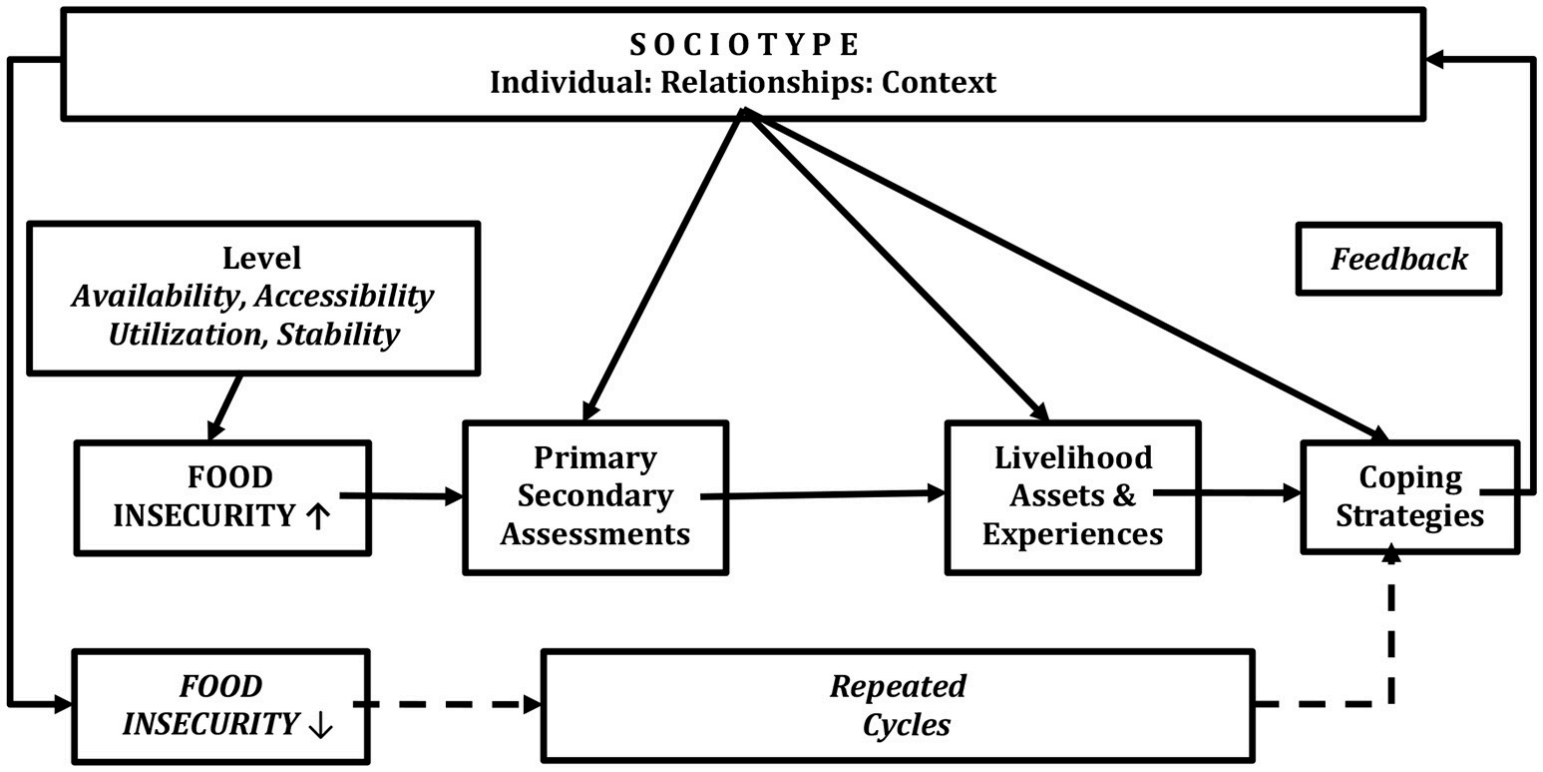
The Sociotype is involved with both the dynamic assessment of the Stresses of Food Insecurity and the Coping strategies to deal with them.

Stress ➜ Assessment I ➜ Strategies ➜ Coping Responses ➜ Successful or Unsuccessful ➜ Appraisal II ➜ Strategy II… (Figure 2).

To deal with FINS, primary and secondary assessments of the stresses come into play. Primary assessment is the immediate cognitive and emotional responses, while secondary assessment takes place later, following more rational considerations. Households/individuals with different sociotypes may respond cognitively completely differently to the same stress, attributable to both their different resources and past life experiences. Given a certain stress, households/individuals with more resources and previous successful coping experiences will probably assess the stress as more controllable than others. A study by Singh and colleagues has shown that perceptions of climate variability and consequent livelihood adaptations differed according to gender and wealth status among the *Adi* community of the Eastern Indian Himalayas. Men and Womens' perception of climate variability depended both on their affluence and on the areas they controlled or worked on. As a result, their coping strategies differed. Wealthy people could adapt to climate variability by intensifying their production systems and receiving more advice and training. By contrast, poor farmers adapted predominantly by diversifying activities (25). At this stage, the Sociotype, representing past life experiences and other environmental factors, works together with other inputs, determining the assessment of the stresses. Assessments are critical to the management of the stress. An extreme case of assessments' role was the different behaviors and outcomes of Holocaust victims when people were under severely deprived conditions and little resources were available for coping. Long-term survivors did not appear to develop many abnormal clinical eating disorders apart from a tendency to hoard food stores (26).

In the coping strategies for FINS, the Sociotype works mainly as a planning framework. Coping strategies could be constructed from one or more Sociotype domains. For example, if a household head gets ill and is not able to work for several months, many strategies, not necessarily mutual exclusive, could be utilized to cope with this stress from the Sociotype framework. In the Individual domain, other household members could work harder (including self-creativity), or collect indigenous edible resources using local traditional knowledge (25), or sell assets, or use barter practice; from the Relationships domain, they may rely on the help from relatives, friends, or use credit; from the Context domain, they may use mutual insurance schemes, or seek for help from formal social safety net schemes, or request technical or short-term financial support from local institutions. Some options may overlap multiple domains. For example, credit could be from relatives or friends (Relationships), or from institutional micro-financial support (Context). Selling assets is a coping behavior from individual household (Individual); however, it also relies on the exchange and market mechanisms of society (Context). Usually autonomous coping strategies (such as barter practice or selling off assets, relying on help from relatives or friends) may improve FINS only at the individual or household level. By contrast, when planned policies involve contextual coping behaviors, e.g., social safety net schemes, these strategies should usually benefit more people. Therefore, the Sociotype is involved in both the assessment of stresses, and in determining strategies necessary to cope with them.

## Resilience and coping in the sociotype framework

Resilience is another concept closely related to coping with stresses of FINS. The definition of resilience by the USAID is the ability of people, households, communities, countries and systems to mitigate, adapt to, and recover from shocks and stresses in a manner that reduces chronic vulnerability and facilitates inclusive growth (27). Resilience building is fundamental to cope with acute or chronic FINS, thus ensuring food and nutrition security (13).

So what is the relationship between resilience and coping with FINS? We would suggest that resilience relates to the *long-term* growth of the coping system to stresses, while coping considers the *short-term* responses to stresses of FINS. These time dimensions are analogous to those of Stability (short-term) and Sustainability (long-term) in the concept of FS (3). Of note, the short-term coping strategies and behaviors may interfere with the long-term resilience building. For example, mining activities in remote areas may temporarily increase local people's income, and adapt to stresses caused by food insecurity. However, unless well-monitored and managed, the subsequent deterioration of the native ecological system may reduce the productivity of local natural biological resources and sustainability, thus having a negative impact on the long term resilience building against food insecurity (28).

The perceived severity and impact of the stress (assessments) (29) and the strategies to build coping strategies and long-term resilience both involve the Sociotype. What differs from the “simple” coping is that, resilience building serves the long-term FS, based on previous experience from dealing with short-term food needs. The dynamic and lifelong-changing sociotype is the constant input of the resilience to FS of households/individuals, which further determines the short-term coping strategies. In other words, the sociotype is another thinking/planning framework to form the strategies to build resilience. A dynamic, consistent and re-iterative feedback exists here as well (Figure 2). This approach, together with Figure 1, may be compared with the model described by Scoones (30) which shows what assets and resources are available for sustainable rural livelihoods. At the same time, the process of building resilience, as part of the new life experiences, continues to reshape sociotype. Resilience is difficult to measure. In order to allow comparability, some quantitative measurements of resilience have been attempted both at the national level (31) and the household levels (32).

In spite of these attempts, building resilience should focus on particular dimensions of FS in specific settings, and the weightings in each dimension would therefore also differ according to circumstances and culture. For example, farmers in rural areas need to diversify their livelihoods apart from agriculture, while people in urban areas have to invest in education to strengthen their potential in job markets; modern societies value social protection nets, while traditional societies respect community cohesion and inter-personal relationships (25). Therefore, a universal assessment of resilience does not exist and qualitative methods are also necessary. The Sociotype, as both an input and planning framework of resilience building, correspondingly differs in various settings, and needs both quantitative and qualitative methods for monitoring and evaluation. There is no one generalizable, normal Sociotype—it depends on the specific situation, FINS stresses cultural values and much more. Therefore, the evaluation of the multi-dimensional nature of the Sociotype requires mixed methodological approaches as detailed in a recent paper (8).

## Coping strategies for food insecurity in the framework of the sociotype

Maxwell has proposed four types of coping strategies: (1) dietary change; (2) increase short-term household food availability; (3) decrease numbers of people eating together; and (4) rationing strategies (33). The limitation of these coping strategies is that they address only short-term food requirements, but not long-term resilience building. A twin-track approach to cope with FINS, addressing both strategies, needs to be considered, using the Sociotype framework.

Table 3 is an attempt of a summary matrix for coping with FINS using such a twin-track approach according to the domains of the Sociotype and dimensions of FS. It is noted that this summary matrix is not an exclusive categorization as some overlap in classification exists. Also, the ecological long-term time fifth dimension of FS, sustainability, is not included in this matrix. This is because the responses to *acute* food insecurity will not, almost by definition, consider the future consequences of relieving the stress. However, at the national level, when planning for *long-term* food security, issues of ecological sustainability will be very important and dealt with under the rubric of Sustainable Food Systems (39).

**Table 3:** Matrix chart of coping responses to food insecurity according to the domains of the sociotype and dimensions of food security.

**Twin-track approach**	**Dimension/sociotype domain**	**Availability**	**Accessibility**	**Utilization**	**Stability**
To address immediate food requirements	Context (National)	1. Food aid (e.g., Food bank)	1. School feeding programs 2. Consume next season's seed stock 3. Cultural: gender role change; eat food that used to be culturally/religiously unacceptable	1. Provide safe and clean water
	Relationships (Household, Family, Community)	1. Small scale irrigation programs	1. Purchase food on credit (34) 2. Borrow food, or rely on help from a friend/relative (34) 3. Restrict consumption by adults in favor of small children/seniors 3. Feed according to working members of HH (34) 4. Send children to eat with neighbors (34) 5. Send household members to beg (34)		1. Food Storage (35) 2. Limit portion sizes at mealtimes
	Individual	1. Food stocks at home (35) 2. Gather wild food, hunt	1. Ration the money to buy prepared food 2. Rely on less tasty andexpensive foods (34)	1. Physiological responses for dealing with semi-starvation (thyroid, adrenal and autonomic nervous systems)	1. Reduce number of meals eaten in a day 2. Periodic fasts
To improve long term food security	Context (National)	1. Establish rural institutions and collective power (Israel Kibbutzim) (36) 2. Ensure access to land (1978 China rural land reform).(36) 3. Asset creation (37) 4. Agricultural Trade Policies (36)	Physical: 1. Invest in rural infrastructure, transport and revive markets (36) Economic: 1. Public work programs (36, 38) 2. Social safety nets (38) 3. Food Stamps 4. Feeding programs (e.g., Brazil) (38)	1. Improve sanitation, health care and education (e.g., latrine construction) 2. Birth Control Programs 3. Ensure food safety e.g., laws and regulations	1. Diversify agriculture and employment (36) 2. Revive access to credit system and saving mechanisms (36) 3. Reinforce infrastructure (36) 4. Insurance scheme for people and assets (36) 5. General price subsidies (lower than market price) 6. Savings and Loan Policies (36) 7. Micro Financing (36)
	Relationships (Community)		Breast Feeding Coaching Pregnancy Banks	1. Immunization programs	1.Mutual insurance 2. Community Planning Committees
	Individual		1. Increase livelihood options (35) 2. Improve working capacity or working skills to increase income (35)	1. Proper food storage and processing	1. Seed reserves

## Case studies of sociotype in action

### Domains: individual, relationships, and context: natural disaster in the pacific islands

Against the background of global climate change, the increased frequency of natural disasters and extreme weather conditions are posing new challenges to FINS issues, particular in island countries. The case with Fiji's cyclone in early 2016 emphasized how ecological sustainability interacts with FINS issues, and equally importantly, showed how *preparedness* is vital for the coping.

In early 2016, Pacific islands including Fiji were hit by the strongest cyclone in the Southern Hemisphere, followed by continuous hurricane and flood. To deal with the shock, the International community responded immediately and provided direct financial, material, and professional support; every affected Fijian family received some money from a national collective reserve pool in the event of natural disasters (Context domain). Food and facilities were stored in the highlands and a distribution network was established before the cyclone, to meet the needs of both short-term feeding requirement and long-term livelihood recovery after disasters (Relationships domain). Individual households were also encouraged to store food and tools before the disaster (Individual domain) (personal communication). From the quick response of the international aid (Context domain) to the food and tool storage and distribution network (Individual and Relationships domains), *preparedness* helped to mitigate the adverse consequences of the cyclone, and to rebuild the FS in Fiji after a natural disaster. How to be best prepared in the future, might be a question that the Sociotype framework helps to answer.

### Domains relationships and context: asset creation in myanmar

The three Sociotype domains are interactive, but not exclusive. Successful copings with FINS must involve comprehensive strategies, which may cover multiple domains, and address both short and long-term demands, such as the assets creation program in Myanmar (37).

Myanmar is the largest country in terms of area in the Southeast Asia. It is rich in agricultural resources, but many Burmese suffer from a high level of FINS, partly because of lack of infrastructure and frequent natural disasters (e.g., floods, cyclones).

Assets creation is a FS project by WFP through building community assets. In slack farming seasons, WFP provides food/cash to community members in exchange for working on infrastructures, including construction and renovation of water schemes, bridges and roads, etc. In the short term, it provides food/cash to affected households for use in hard times; in the long term, the assets built will increase the resilience of the community.

Assets creation programs in Myanmar cover all the three Sociotype domains. From the context domain, the coping strategies strengthened infrastructure, thus hopefully mitigating the impact of future disasters; they also increased access to markets by constructing and renovating roads, thus reviving the function of markets. From the relationship domain, community's cohesion was strengthened by working together toward the common interest. From the individual domain, food/income sources in participants' families were diversified, thus reinforcing the household's resilience. This case showed how the strategies in the Relationships and Context domain affect households/individuals, though the up-scale Relationships and Context domains in an ecological model are not as visible as the down-scale Individual domain. All the strategies work together as a system, bringing the household/community to an overall higher degree of FS. If the potential stresses of FS returned, because of the created assets in a community and its previous experiences in combating FINS, community members would assess the threat differently and frame coping strategies based on the current reshaped Sociotype.

The case study in Myanmar was driven first by international aid, which is a resource from out of the system. In some settings, structural changes in the system itself may also bring influential positive impacts.

### Domain context: land reforms in east asia

After World War II, the succession of land reforms in some East Asian countries, e.g., China, Japan, and South Korea, allocated agricultural land to individual households, and established supportive rural institutions in financial, marketing, agriculture technical training, etc. (40). These structural changes solved most of the hunger issues in these countries and areas, and they also consolidated the basis for further industrial development. The rural land reform in China from 1978 epitomized this transformation. Started from the privatization of land utility reform in the Context domain, it triggered down-stream changes in the Relationships and Individual domains. In the former, households from sole family units became independent production units as well. This composition of production unit helped to maximize the collective power of household members. In the latter, household members were motivated to utilize agricultural technology and work carefully on their own lands. This case example shows the fundamental role of the Context domain in the Sociotype ecological model, only through which resilience to FINS and long-term FS may be achieved.

## Future challenges

Can one teach coping skills for food insecurity? This question has to be tackled at different planning stages depending on the country or region involved as well as the culture, education and socio-economic level of the population. Nationally, there must be policies for emergency situations including international aid, storage and distribution facilities. At the household level it probably can only be dealt with over the short time scale. Where possible, households should have stores of food and money. Also, home grown produce (mainly rural, but urban gardens are developing) and cooperation and food sharing between family and neighbors will be helpful (41).

A surrogate marker for chronic food insecurity is chronic hunger. Ideally, no child should go to bed hungry (5). If this happens, then there is a breakdown in family duties, public health monitoring, and national values. Food is a human right which is recognized in article 25 of the Universal Declaration on Human Rights and in article 11 of the International Covenant on Economic, Social and Cultural rights: long-term sustainable food security will ensure it (42). Ensuring Food security is everyone's interest and responsibility. The Sociotype ecological framework allows planning strategies for coping the food insecurity stress at the national, household and individual levels.

## Author contributions

EB conceptualized the idea. EB and WP drafted the manuscript. SD undertook extensive critical review of the manuscript. All authors finally approved the submission of the manuscript.

### Conflict of interest statement

The authors declare that the research was conducted in the absence of any commercial or financial relationships that could be construed as a potential conflict of interest.
